# Digital Image Correlation and Finite Element Computation to Reveal Mechanical Anisotropy in 3D Printing of Polymers

**DOI:** 10.3390/ma15238382

**Published:** 2022-11-24

**Authors:** Sofiane Guessasma, Hedi Nouri, Sofiane Belhabib

**Affiliations:** 1 INRAE, BIA Research Unit, UR1268, Rue de la Géraudiere, 44316 Nantes, France; 2 Laboratory of Electromechanical Systems (LASEM), National Engineering School of Sfax, University of Sfax, Sfax 3038, Tunisia; 3 Laboratory GEPEA, CNRS UMR 6144, Department of Mechanical Engineering, Institute of Technology, Carquefou Campus, Nantes Université, Oniris, F-44000 Nantes, France

**Keywords:** fused deposition modelling, digital image correlation, acrylonitrile butadiene styrene, mechanical testing, printing orientation

## Abstract

In this study, we propose to revisit the mechanical anisotropy inferred to printed ABS polymers using fused deposition modelling by combining digital image correlation (DIC), mechanical testing and finite element computation. Tensile specimens are printed using different design orientations and raster angles. Monitoring of deformed samples is performed, and strain fields are derived for each configuration. Finite element modelling of the 3D-printed material behaviour is considered to shed more light on deformation mechanisms. Experimental results show that a heterogeneous strain field develops, leading to more significant strain localisation for samples printed with the main dimension aligned with the building direction. The optimal printing angle allowing the filament to be crossed at −45°/+45° shows the best behaviour with even larger elongation at break compared to the raw material. However, digital image correlation based on optical imaging shows that a limiting scale exists for revealing the effect of filament orientation on strain localisation. Finite element results reveal the nature of the strain localisation as related presence of porosity close to the frame and the development of asymmetrical filling within the printed structure.

## 1. Introduction

Additive manufacturing (AM) opens a window into a new way to process materials using 3D digital models [1]. In opposition to classical machining processes involving material removal, AM offers versatile 3D-printing approaches that generates a smaller amount of material waste [2]. It also shortens the fabrication process into a main step of tool path generation, incidentally reducing design dependence on tooling [3]. According to the state of the processed material, categories of AM can be defined [4,5,6,7]: liquid for fused deposition modelling [8,9], stereolithography [10], Polyjet [11,12], solids for laminated object manufacturing [13] and powders for selective laser sintering [14], laser powder forming [15,16], etc. These processes involve different physics of bonding to build the part resulting from sintering, melting, photopolymerization or deposition [7]. Despite these differences, all these processes share the same idea of sequentially building the design by adding droplets, filaments or layers [17].

AM has a growing spectrum of applications reflected by the trend in AM unit sales [18]. These applications concern key sectors such as biomedicine and more particularly, tissue engineering [19] and prosthetics [20], the automotive industry [21], aerospace technology [22,23] and with a wider market share, the consumer goods market. 

Fused deposition modelling, or FDM, is one of the most versatile techniques. It allows the production of complex geometries using controlled amounts of materials with a lesser dependence on tooling [2,3]. Consequently, manufacturing costs are reduced compared to traditional processing technologies. In addition, FDM is one of the most popular 3D-printing processes, probably because of its ease of use and the increasing number of available materials that can be processed.

Due to the fact that the performance of the parts manufactured using FDM are sensitive to the building direction, several contributors have tackled the effect of build orientation [24]. These efforts have led to a better understanding of the effect of sequentially adding layers of the fused material on the generated mechanical anisotropy [25,26]. For instance, Panda et al. [27] developed a neural computation approach to study the combined effect of part orientation, layer thickness and raster angle on the mechanical performance of 3D-printed ABS material. The authors found that largest mechanical strengths are observed when the orientation is selected at the highest level. Porosity is found to play a key role in the lack of mechanical performance and, more particularly, pore connectivity [25]. A recent study by Dawoud et al. [28] compares the mechanical performance of samples manufactured using FDM and injection moulding. The authors show that material compaction explains the better results of injected samples. They also conclude that, among the set of FDM process parameters, adjusting the raster gap to negative levels helps to improve the performance of the printed parts.

An earlier study by Sood et al. [29] reports on the effect of a larger number of FDM process parameters, namely layer thickness, orientation, raster angle and width, and the air gap. The authors report a deterioration of the mechanical performance for thick rasters, as was already pointed out by Shubham et al. [30]. A much earlier study by Ahn et al. [31] demonstrates the significant anisotropic effect of raster orientation on the tensile performance of FDM-based samples. In particular, Smith et al. [32] point out differences in stress–strain trends between polycarbonate tensile specimens printed using three different building orientations. Their result matches the fact that the worst printing orientation takes place when the building direction is aligned with the loading direction [31]. Panda et al. [33] developed a computer-based method to measure the volume error generated by the selection of the build orientation. The authors showed that adopting such approach can optimise the part orientation with regards to dimension accuracy. 

The physical explanation of the lack of performance due to the layering effect can be attributed to the thermomechanical history of the processed part involving sintering phenomena [34]. In fact, with FDM processing, there is a difference in temperature between printed layers. This difference causes stress accumulation, and consequently, damage or delamination may appear under low loading levels. This explains several reported drawbacks of FDM such as the negative effect of a large layer thickness on tensile performance [29,30], and the dependence of geometrical accuracy on liquefier temperature [35].

If the building direction is largely credited with tailoring the performance of printed materials, filament crossing in the plane perpendicular to the building direction takes the other part of the credit. The laying-down characteristics in this plane are represented by the raster angle. The best tensile performances are obtained when filaments are crossed in a sequence of −45°/+45° [28]. The general idea is to adapt both orientation and raster angle. To build through the lowest dimension (i.e., building direction aligned with part thickness), Ahn et al. [31] reported that a raster angle of 90° is not a good choice for tensile properties since the loading is only taken by the inter-filament bonding instead of the filaments themselves. The combined choice of the raster angle and the orientation must also take account of the travel distance of the filaments. Sood et al. [29] reported a negative effect of small raster angles on both tensile and flexural strengths. The authors attribute this effect to stress accumulation during processing and the resulting distortion taking place with large rasters.

Although the cited literature helps us to understand the effect of process conditions on the performance of printed parts, the overall picture is incomplete. We take as proof the fact that all of the former studies focus on overall mechanical response and derive overall macroscopic correlations with respect to the process conditions. In this study, we propose to address the strain heterogeneity that takes place during tensile tests using digital image correlation [36]. This method has been used to derive the triaxial strain field in 3D-printed ABS-based composite [37]. This method explores submacroscopic effects, which are useful to explain the deformation mechanisms taking place at a scale much lower than the macroscopic one, i.e., filament diameter or inter-filament space. Digital image correlation represents a new trend, making it possible to take greater advantage of the mechanical test by recording the specimen deformation during the mechanical test [38,39]. Some of the main advantages of the DIC as a non-contact technique is the access to the full field of the specimen deformation without gluing strain gauge, the relative ease of implementation, and being cost-effective if open source solutions are used. This technique is used in this study to reveal the extent of inhomogeneous deformation of printed ABS subjected to tensile loading under various printing conditions. DIC results are discussed by considering the effect of the raster angle and the build orientation. In addition, the experimental results are compared to finite element predictions which are based on the implementation of the filament trajectory and process-induced porosity.

## 2. Materials and Methods

### 2.1. Materials

The feed material is a thermoplastic polymer: ABS, or acrylonitrile butadiene styrene, with the chemical formulation, (C8H8)x-(C4H6)y(C3H3N)z). ABS is purchased in the form of an extruded filament of 1.75 mm in diameter. Its glass transition temperature is 108 °C [26]. The elastic properties of ABS are referred to as P0000. A support material is needed for some printing configurations to prevent design collapse during the printing process. As stated in the uPrint SE product specifications, the support material is SR-30 soluble polymer. 

### 2.2. Sample Preparation

**Figure 1:**
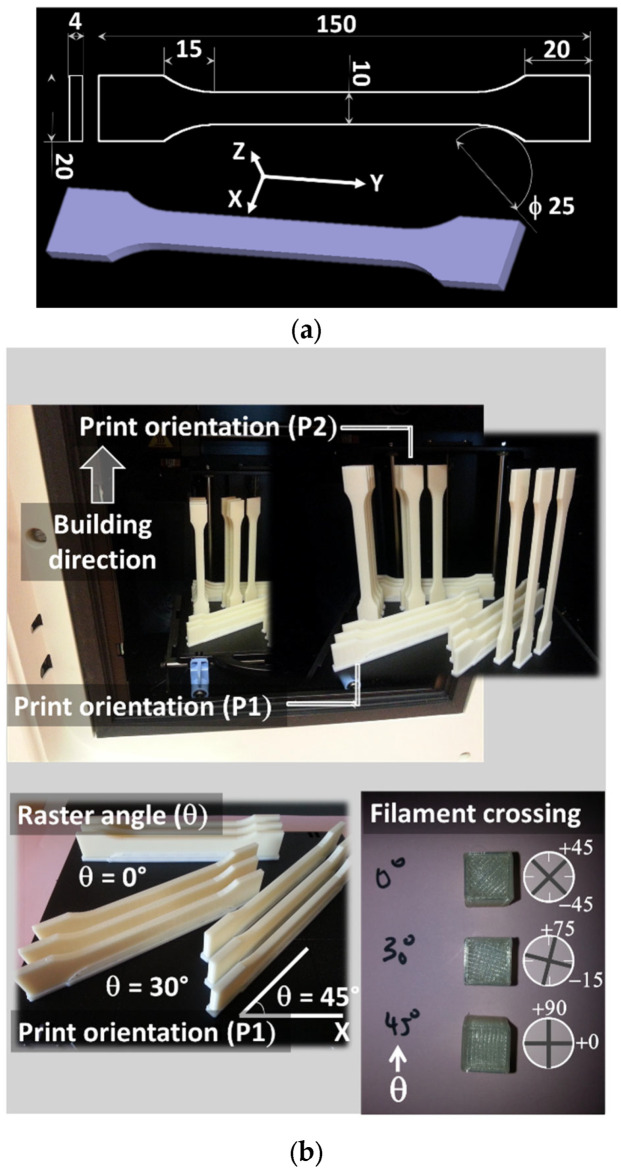
(**a**) Geometry and main dimensions of the tensile specimens in mm; (**b**) definition of FDM print parameters used to assess anisotropic effects of ABS tensile specimens.

Design geometry is a typical tensile specimen with dimensions adapted according to EU Norm ISO 527-2 [40]. Sample dimensions are 150 × 10 × 4 mm^3^, with a gage length of 56 mm. The tessellated geometry of the tensile specimens is performed to obtain (Standard Tessellation Language) STL files, which are further converted into tool path trajectories using CatalystEX slicing software from Stratasys. A large number of parameters need to be defined for FDM. The selection of these parameters is based on previous studied by the research group [25,41]. For instance, two main filament diameters are used: 1.75 mm and 3.00 mm. However, the choice of using a filament diameter of 1.75 mm comes from the fact that nearly 80% if the FDM market is based on this diameter. The detailed printing parameters are summarised in Table 1. Tensile specimens are printed using an uPrint SE printer from Stratasys, Valencia, CA, USA (Figure 1a). Six different printing configurations are considered by combining three levels of raster angles and two printing orientations (Table 2). Each raster angle generates a different filament crossing sequence, as shown in Figure 1b and Table 2. All raster angles are selected below 45° because of the symmetry of the filament crossing. The axis of symmetry being 45°, an angle above 45° reproduces filament arrangements similar to 90°—the prescribed angle. For instance, the angle of 60° provides the same filament arrangement as 30°.The angular spacing between selected raster angles reflects meaningful variations in the mechanical response. More information about the raster angle definition and selection can be found in [42].

In order to easily remove the support material, a smart filling option is used. After printing, there is no need for a cleaning step since the samples are easily detached from the support base.

### 2.3. Mechanical Characterisation

**Figure 2:**
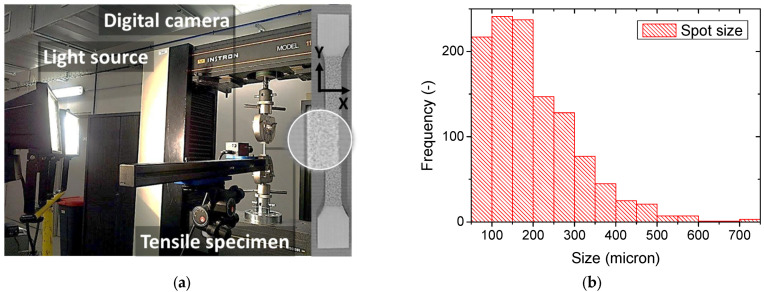
(**a**) Experimental setup composed of a digital image correlation device and tensile testing of ABS samples manufactured using fused deposition modelling; (**b**) spot size distribution of the speckle used for DIC measurement.

Tensile tests are conducted at room temperature using the INSTRON Model 1185 testing machine (UK) with a load capacity of 1 kN (Figure 2). Tests are performed using a crosshead speed of 5 mm/min, corresponding to a strain rate of 0.001 s^−1^. All tests are conducted at 23 °C and 50% RH. Five replicates are used for each printing configuration, making it possible to derive average engineering constants according to the ISO standard, 527-2 [40].

Young’s modulus, yield stress, elongation at break and tensile strength are derived for each printing configuration. All tests are conducted until complete failure of the specimen. 

The tensile test is synchronised with a digital image correlation device developed by the Correlated Solutions company at the University of South Carolina (Columbia, SC, USA). This device is composed of a 4 megapixel (2000 × 2000 pixels) full-frame camera, a light source and VIC-2D control software. The frame rate is adjusted to five images per second and the dimensions of the ROI (Region of Interest) are selected to be as large as possible (50 × 8 mm^2^) to cover the gauge area. 

Prior to testing, the surface facing the camera is coated with a layer of speckle resulting in a surface texture with a large dispersion of the spot size, reaching small spots below 77 µm microns and exhibiting an average spot size of 200 microns (Figure 2). Speckling is performed as a two-step protocol, starting with a uniform white coating, which is dried before applying a layer of black paint on top of the first layer of paint. Surface measurements of displacement fields are obtained from the ongoing optical test recording using VIC-2D software. The digital image correlation is based on subsets of 15 pixels in size with a step size of five pixels, where the physical size of one pixel is 15 microns. A filter size of 15 pixels is used to avoid noisy strain. This recommended value makes the virtual strain gauge size as large as 75 pixels. The subset size is three times greater than the lower limit tolerated by the DIC code, and the step size is equal to the minimum value that makes it possible to resolve the speckles. The minimum subset size/step size ratio recommended for a fine DIC result is 9/5. The one used in this study is almost 1.7 times larger. The subset dimensions are selected as low as possible in order to study the effect of the filament raster. These dimensions are, however, comparable to the average spot size of the speckle and may lead to DIC failure. To ensure that displacement errors are accounted for, the DIC code uncovers the regions where displacement components are not found and refers to these regions as “holes”. Preliminary tests show that DIC still captures the displacement field within the full ROI and neither measurement errors nor holes are detected. This can be attributed to the fact that a large proportion of the spots representing 61% of the total population within the numeric distribution (Figure 2a) are below the subset size and resulting in a relative dense speckling.

In order to scale the measured tensile properties with respect to the feed material, supplier data crossed with tensile experiment results are used. Fracture scenarios are qualitatively evaluated using a Philips SEM 525 M scanning electron microscope with two operated acceleration voltages at 10 kV and 15 kV. Image resolution is adjusted to 1280 × 960 pixels. Observations are performed at different magnifications ranging from ×10 up to ×100, allowing for a pixel size of 0.91 to 4.63 microns. Fracture surfaces are coated using gold prior to SEM observation.

## 3. Finite Element Computation

The purpose of the finite element computation is to provide a support for the interpretation of DIC results based on the implementation of the sliced geometry. Because one camera is used for DIC, only surface strain effects can be captured, and even for the measured strain patterns, some of the measurements cannot be properly interpreted if the effect of the third dimension is not considered. Finite element simulations provide predictive capabilities based on out-of-plane filament trajectory information. All computations are performed using Ansys software. The geometry is imported from the sliced model considering a limited length (eight layers for a total length of 2 mm) to maintain a reasonable model size. The model implementation considers the conversion of the filament trajectory provided by the slicer into a 3D model and adding the filament in-plane dimension according to the nozzle dimeter. The material properties are adjusted to ABS material by considering an isotropic elastic model. This material model is defined by an elastic modulus (2310 MPa) and Poisson’s coefficient (0.35) and referred to as the condition P0000 (Table 3). The raster angle is considered directly from the gcode by converting the trajectory into a skeleton as mentioned earlier. The filament arrangement is thus directly implemented according to the selected raster angle. The thickness of the skeleton is added based on the selected filament diameter, which corresponds to the nozzle size. The porosity generated by the process is also considered. Indeed, the material laying down is handled by building the geometry from the gcode, which leaves space between adjacent filaments. This space represents the process-induced porosity in fused filament process. Meshing is performed using tetra elements with three degrees of freedom per node. The model size is varied to conduct a mesh sensitivity analysis. The model size varies from 6285 to 6.18 × 10^5^ dofs (degrees of freedom). The element size varies in the irregular meshing to cope with material discontinuity. The average element size range is between 63 µm and 290 µm. Tensile boundary conditions are used. An isotropic elastic material model is considered based on experimental data. The linear elasticity problem is solved using an iterative process based on a pre-conditioned conjugate gradient solver. Strain results are derived for discussion purposes as well as error in the prediction of macroscopic quantities such as Young’s modulus.

## 4. Results and Discussion

### 4.1. Experimental Evidence

**Figure 3:**
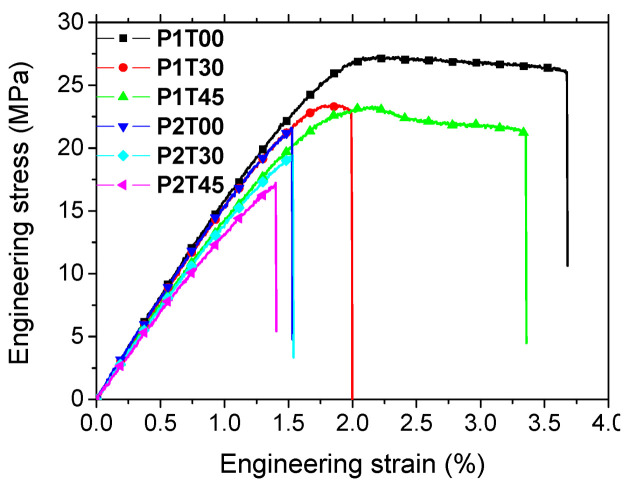
Tensile response of FDM-based ABS specimens as a function of orientation (PX) and raster angle (TYY).

Figure 3 shows the tensile response of ABS samples for all attempted printing configurations. The highest trends correspond to printing configuration P1 combined with the minimum raster angle (θ = 0°). Notable differences appear to be more related to the printing configurations than to the raster angle. These correspond to wider nonlinear branches for P1 compared to short abrupt failures close to the yield for P2. Secondary differences are related to the choice of the raster angle with higher trends being correlated to decreasing values of θ.

Mechanical parameters representing all stages of deformation are derived from the reading of the force displacement results and summarised in Table 3. The magnitudes for all configurations are based on statistics performed on five replicates. Poisson’s coefficient results show no clear trend. When compared to the magnitude of the wire (0.35), the change in Poisson’s coefficient fluctuates between −5% and 20%. This change is measured as the relative difference, in percent, between Poisson’s ratios of the neat material ($\nu_{n}$) and the printed one ($\nu_{p}$).

(1) $$\Delta \nu_{p}\left(\%\right)=100\times \left(\nu_{n}-\nu_{p}\right)/\nu_{n}$$

The relative change in the magnitude of the other engineering quantities is derived in a similar way. Any negative change in the mechanical properties is referred to as a loss and the sign is simply dropped.

Stiffness loss related to FDM processing evolves between 27% and 41% with respect to P0000 depending on the printing orientation and raster angle. The closest orientation to the actual material is P1, with −31% compared to −37% for P2. These differences are, however, not sufficient to infer a strong orientation effect on stiffness. Yield stress values for ABS are generally not documented by the suppliers. In this study, we obtained 30 MPa as an average. When compared to the performance of 3D-printed samples, yield stress loss is limited to 25% for P1 and 37% for P2. Tensile strength of ABS is reported by several suppliers to be in the range of 34–51 MPa with an average of 42 MPa. Tensile measurements made by the authors on ABS wire show that the material used for printing lies within the lower limit, where the ultimate engineering stress corresponding to the engineering stress at the rupture point is confused with tensile strength. Technical data sheets do not report differences between tensile strength and ultimate engineering stress for ABS although we obtained slight differences (<2 MPa excluding P1T30) by 3D printing due to the mechanical necking before rupture. The largest tensile strength of 28 MPa reaches −18% of the ABS wire tensile strength. On the average, 3D printing results in a tensile strength loss of about 35%. In this case, the same orientation effect is depicted with slightly larger magnitudes (−27% for P1 and −47% for P2). These results are in line with the various reported trends [24,28,29,31]. The correlation between tensile strength and raster angle is hypothetically reported by Sood et al. [29] as being related to the filament distortion effect involving phase shrinkage and residual stress accumulation. Dawoud et al. [28] demonstrate that the best performing configurations are those where the layers are forced to lie in the plane containing the loading direction (printing configuration P1). These differences can be attributed to distinct bonding behaviour in the building direction (low cohesiveness between successive layers) compared to the bonds generated by inter-filament crossing within the built plane. 

**Figure 4:**
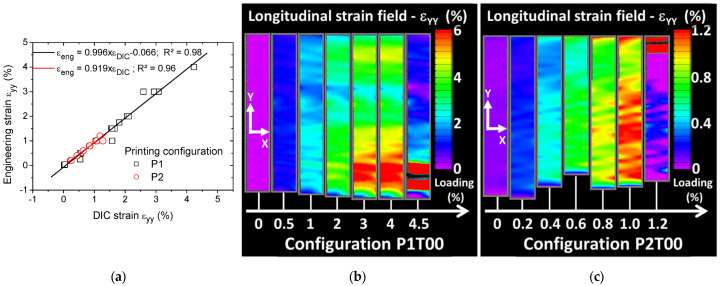
(**a**) Comparison between engineering strain and longitudinal DIC strain as a measurement of DIC accuracy. Longitudinal strain field as a function of the loading level of FDM-based ABS polymer samples obtained using digital image correlation. Comparison between printing configurations (**b**) P1 (best performing) and (**c**) P2 for a raster angle of 0° (raster sequence +45°/−45°).

**Figure 5:**
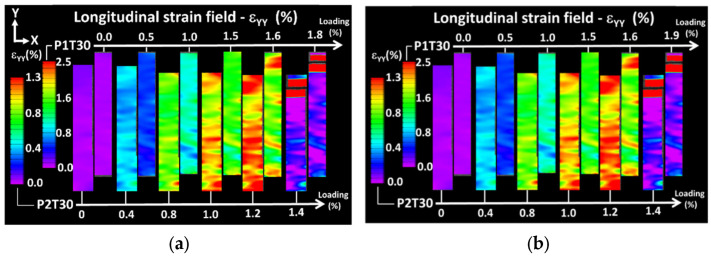
Complementary longitudinal strain field evolutions resulting from digital image correlation comparing the effect of the printing configuration of FDM-based ABS polymer specimens for two raster angles: (**a**) θ = 30°; (**b**) θ = 45°.

**Figure 6:**
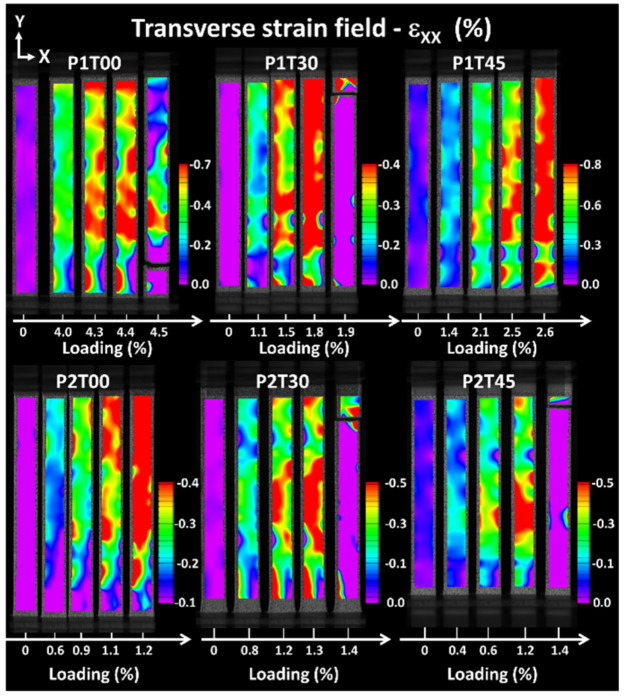
Comparison between transverse strain fields resulting from digital image correlation highlighting differences between printing orientations P1 and P2 as a function of raster angle θ.

**Figure 7:**
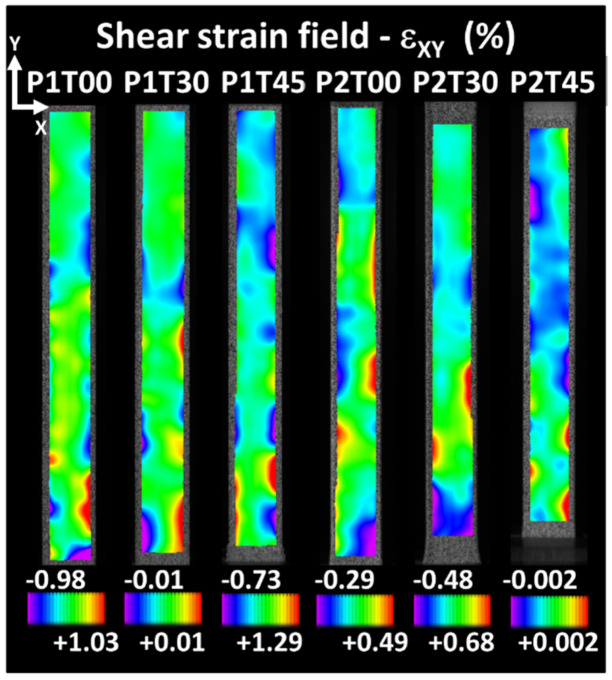
Shear strain fields as a function of printing orientation and raster angle θ.

Concerning the elongation at break measured as the largest cross-head displacement before rupture, the material data sheet provides a huge range for the raw material, typically between 1% and 57% depending on the ABS grade. Our own measurements show that the elongation at break of the feedstock material is limited to 2.93%. This is the only parameter where a positive effect of orientation is obtained. Indeed, the elongation at break results in Table 1 reveal that only 10% of loss in elongation at break is due to printing orientation P1 with a positive effect of 37% obtained for P1T00. A strong negative effect for printing orientation P2 is reported here with a magnitude as large as −49%. The evolution of the strain fields derived from DIC is presented in Figure 4, Figure 5, Figure 6 and Figure 7 for all printing conditions. Before detailing the observed effects, the quality of DIC measurements is first discussed. Although, the average subset size is nearly comparable to the average speckle size, the derived strain fields contain no holes. This means that DIC succeeded in finding the displacement field despite the low limit for subset size. This can be explained by the relative low strain experienced by the printed specimens. As shown by Yaofeng and Pang [38], the average speckle size is only a global indicator of the speckle quality. The authors instead proposed the concept of subset entropy to better capture the quality of the speckle. Schnittker et al. [37] discussed the relevance of high-contrast speckle patterns to achieve successful DIC results. Hassan et al. [39] suggest using a varied subset size to adapt the tracking function to the variability in the speckle size. In this study, the variability in the speckle size provided in Figure 2 demonstrates that tracking points on the speckle pattern are possible because of the presence of larger spots. However, this does not mean that the accuracy of the DIC strain field is guaranteed. This accuracy is derived by comparing the average strain on the full ROI to the engineering strain measured from the Instron machine at different load levels, as shown in Figure 4a. Although there are several approximations made here such as the similarity between the true and nominal strain and the relevance of the strain of the ROI with respect to the full sample, the result of DIC is still satisfactory. A slope of nearly one is obtained for both printing configurations with a low dispersion. Differences between DIC and engineering strain levels are, on average, 12% and 10% for P1 and P2 printing configurations.

Figure 4b,c show the evolution of the longitudinal strain field for an increasing loading level up to failure. Full sequences of longitudinal strain evolutions are provided as supplementary videos. Two printing configurations are illustrated in Figure 4, namely P1T00 and P2T00 with the same raster angle of 0°. The common point between the two studied configurations is the various spots of high strain positioned perpendicularly to the loading direction. Two different scaling of the strain levels are used for the two conditions. The strain value is provided as a percentage for ease of reading of the data. The strain field evolution shows that these spots originate from lateral edges and extend transversely toward the centre. The observed field is far from reflecting the behaviour of a homogeneous material. Differences appear between the printing configurations P1T00 and P2T00 related to the magnitude of the strain and the extent of localisation. For P1T00 (Figure 4b), strain localisation near the fixture reaches levels higher than 6%. For P2T00 (Figure 4c), larger regions of strain localisation appear at much lower longitudinal strain levels of 1.2%. This indicates a stronger and faster damage trend for the worst printing configuration connecting transversely. It is also difficult to directly link the observed strain fields to the filament crossing configurations because filament ends are wrapped by a rectangular frame prior to layer building. This external frame is meant to confer a mechanical stability to the raster. In addition, the direct signature of layers is not observed because of the relatively low resolution of the optical system used for DIC. Figure 5 shows the longitudinal strain fields for the remaining raster angles. Two different scaling of the strain levels are used for P1T30 and P2T30. The strain values are also provided as a percentage. 

The comparison between printing configurations P1 and P2 confirms the same differences about the extent and magnitude of strain localisation. The strain magnitudes reach different levels compared to the former cases (1.4% for P1 compared to 2.5% for P2). In addition, strain localisation occurs at lower strain levels for θ = 45° compared to θ = 30°. This observation is expected from the reading of the mechanical responses in Figure 3. 

Figure 6 compares the transverse deformation fields for all printing configurations. Because of the differences in strain magnitudes expected for each printing condition, scaling of strain level is adapted according to each condition. This adjustment allows to capture the strain heterogeneities for each printing condition. The negative levels obtained result from the transverse contraction associated with tensile loading.

Larger strain levels are depicted for all raster angles associated with printing orientation P1. Figure 6 also shows that the lateral contraction remains heterogeneous up to the failure point for both printing orientations.

Differences in strain levels between printing orientations P1 and P2 cannot be related through DIC to differences in filament paths because of the low resolution in reading filament deformation.

Figure 7 depicts the shear strain field for an engineering strain of 1% for all considered conditions. For each condition, the strain scale is adjusted to highlight the strain heterogeneity, which corresponds to a total of six printing configurations.

The main feature revealed is the existence of strain localisation, which develops at the lateral edges. The heterogeneous strain field does not considerably evolve from the views shown in Figure 7, except for the bound values that are stretched as the load continues. The highest shear strain levels obtained with each printing configuration similarly depend on the former strain fields on the printing orientation and raster angle. For all printing configurations, the levels of shear strain remain lower compared to transverse and longitudinal strains.

It can be stated that DIC provides valuable surface information about the effect of the printing conditions, which materialises through the observed differences in terms of strain levels intensity and localisation behaviour. The observed effects are retrieved fast enough to capture the entire strain field related to the overall mechanical response, proving that the mechanical anisotropy can be reasonably derived. However, the resolution is not sufficient to relate the localisation behaviour to the filament scale. There is thus a need to retrieve such missing link using a complementary technique such as finite element computation that implement the filament trajectory. The outcome of this technique is discussed in the next sections.

**Figure 8:**
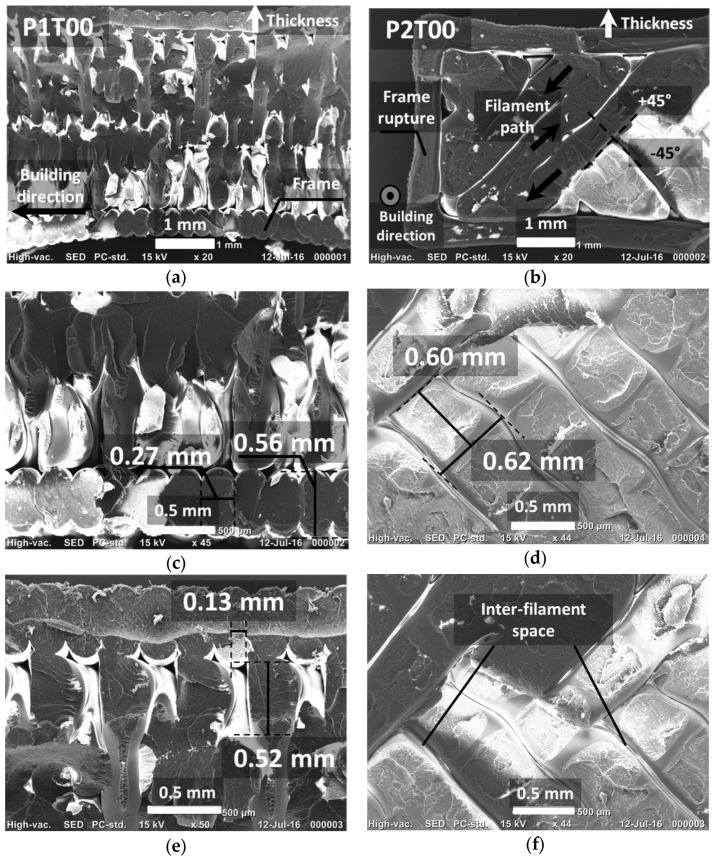
SEM micrographs at different magnifications showing geometric characteristics of fracture surfaces of best and worst performing printing configurations, P1T00 and P2T00. (**a**) P1T00—full view across the thickness, (**b**) P2T00—full view across the thickness, (**c**) P1T00—raster view, (**d**) P2T00—raster view, (**e**) P1T00—upper view, (**f**) P2T00—zoomed view.

Qualitative evaluation of the damaged samples is further explored using SEM micrographs in Figure 8. Fracture surfaces of the two printing configurations are compared for a raster angle of 0°, including the best performing condition (P1T00) and the homologous one for P2 (P2T00). Both printing configurations show common characteristics of ductile rupture. The main difference between the two configurations is the presence of large, fractured surfaces in P2T00 compared to more disconnected fracture spots for P1T00.

**Figure 9:**
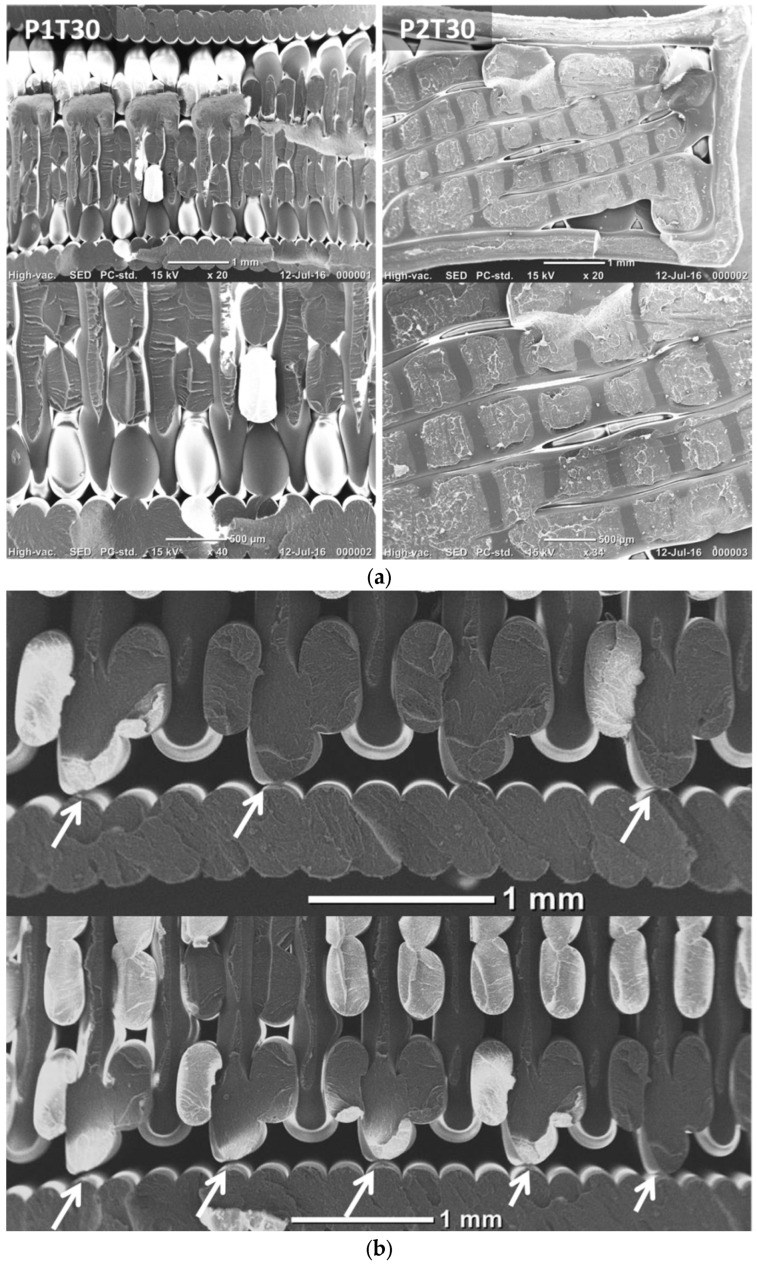

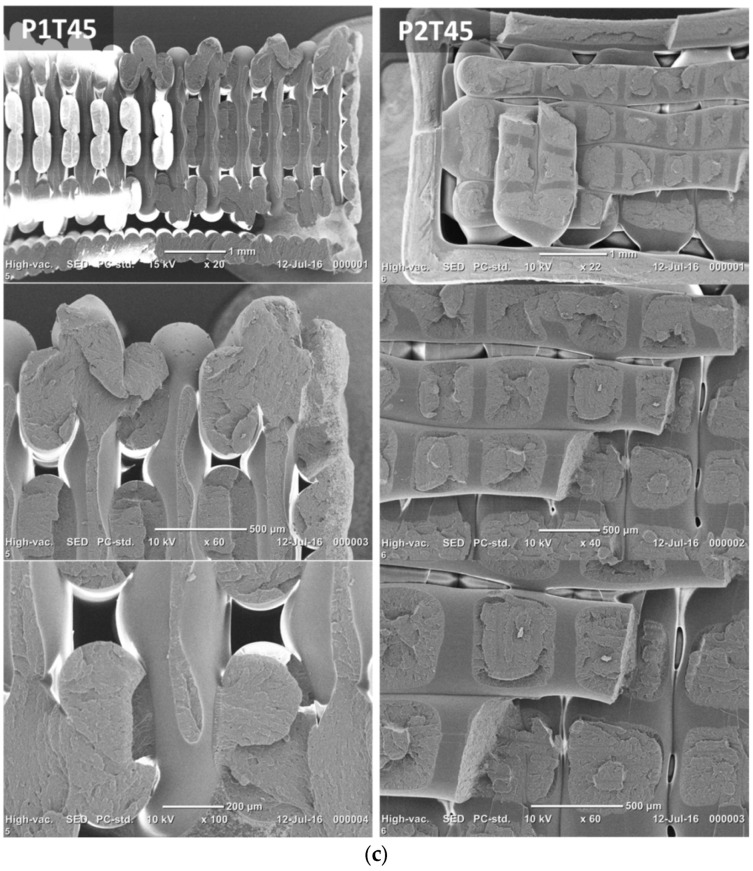
(**a**) SEM micrographs of fracture surfaces showing the effect of raster angle θ = 30°; (**b**) magnified views showing the decohesion between the frame and the raster; (**c**) SEM micrographs of fracture surfaces for θ = 45°.

For the latter configuration, filaments forming the external frame appear to be more cohesive and not particularly affected by the normal loading. These filaments are fractured transversally but with no evidence of inter-filament decohesion. P2T00 shows signs of inter-layer rupture, which can be seen by the ruptured frame at different locations. Despite the clear presence of a network of inter-filament spacing, the extent of inter-filament decohesion is not significant. It appears here that layer debonding is the damage mechanism that leads to the rupture in P2T00. Figure 9 shows the SEM micrographs of the fracture surfaces of the other printing conditions. Figure 9a compares the fracture characteristics for θ = 30°. In addition to the previous comments, which still hold here, there is decohesion between the frame and the remaining part of sample P1T30.

This decohesion is not to be confused with the gap naturally present between the frame and the raster, as will be further detailed in the next section. It concerns, as illustrated in Figure 9b, those sites where there is a limited contact between the frame and the raster. When loaded, these contact points are subject to tension, and decohesion occurs at the particular positions indicated with arrows in Figure 9b.

The homologous condition P2T30 shows inter-filament decohesion that occurs along the width of the specimen in addition to layer debonding. This additional damage mechanism may explain the lower ultimate performance of P2T30 compared to P2T00. The worst performing conditions from each printing configuration (θ = 45°) are compared in Figure 9c. The tendency of a frame decohesion is confirmed with P1T45. A larger inter-filament space also indicates a lower cohesive structure during loading. For P2T45, the strongest layer debonding observed is also complemented by a lack of filament cohesion materialised by the large spacing between filaments and reaching 160 microns in some regions.

### 4.2. Discussion of Main Effects

**Figure 10:**
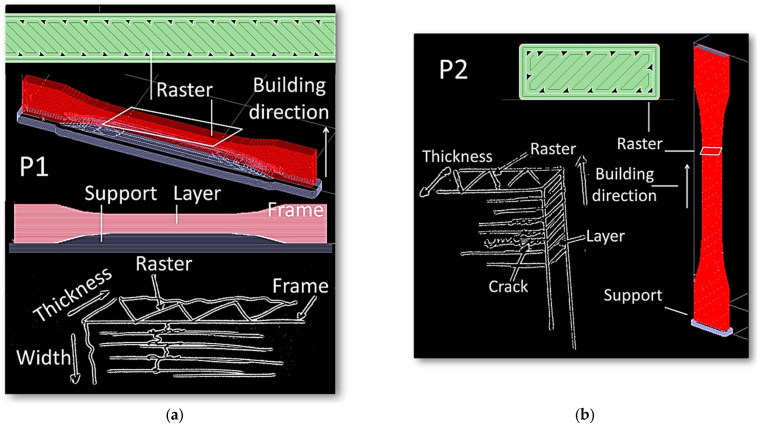
Mechanisms of strain localisation illustrated for printing configurations (**a**) P1 and (**b**) P2.

DIC results show differences in strain localisation that explains the overall mechanical behaviour. Since this localisation is not fine enough to reveal the role of the filament arrangements, complementary information is brought from both the analysis of SEM micrographs and the slicing configurations reported for each printing condition. The examination of the strain localisation at the edges for both the P1 and P2 configurations calls for particular damage mechanisms that are qualitatively revealed using SEM micrographs and further detailed in Figure 10.

In the first case (printing configuration P1), the presence of a particular type of stress concentrator, namely the porosity close to the frame, plays a central role in precipitating the failure of the specimen. As suggested in Figure 10a, filaments are first deposited along the boundaries of the specimen to achieve a better mechanical stability. As a result, there is a substantial change in the filament trajectory close to the edges, which, in turn, results in particular types of porosities that have a triangular form. Three-dimensional views of these porosities reveal a much higher complexity, as suggested in a former study [41]. It is believed that such porosity triggers the failure of the specimen through specific cracking behaviours. For the printing configuration P1, large deformation of the frame close to the triangular porosity is expected due to the lack of cohesiveness. These preferred sites for cracking are illustrated in Figure 10a. Subsequent crack growth along the specimen width and normal to the raster is more or less disturbed by the layering effect. For the printing configuration P2, a similar mechanism is expected but more particularly involves the weak cohesion between filaments in the building direction. The cracks probably begin at the inter-filament space and then connect to the frame porosity, travelling across the width of the specimen.

**Figure 11:**
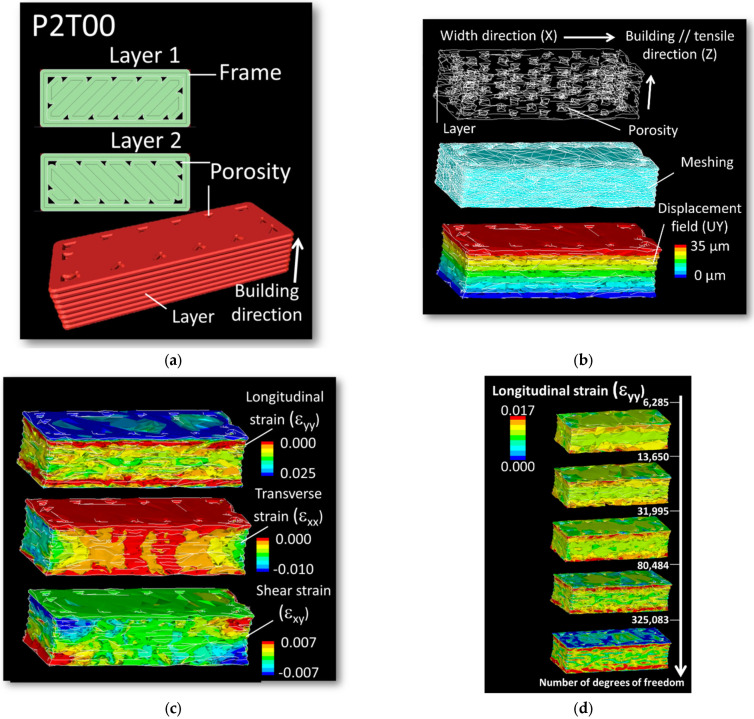
(**a**) Asymmetry in filament trajectories and generated porosity for printing configuration P2T00; (**b**) related finite element model including the geometry, meshing and load conditions; (**c**) predicted strain components highlighting the development of shear strain as a result of asymmetry in process-induced porosity; (**d**) mesh sensitivity analysis revealing the quality of the longitudinal strain field.

Regarding the shear strain distributions depicted in Figure 7, these can be interpreted as being related to the misalignment between loading and building directions for printing configurations P1. However, for printing configurations P2, the presence of shear strain is more difficult to understand. Indeed, the presence of such a strain component for printing configuration P2 is not obvious to understand. The reason is, in fact, related to the asymmetry within the raster itself. If we look at two successive cross-section views generated by the tool path, the beginning and end of the filaments makes it impossible to achieve symmetrical filling of the part (Figure 11a). In configuration P2T00 shown in Figure 11a, the building up of the part is mainly composed of the alternation of two main layers (layer #1 and layer#2) along the length of the sample. A closer look at these layers reveals that the porosity generated near the frame is not symmetrical. Unfortunately, DIC cannot show the effect of inhomogeneous porosity within the core of the specimen. To better illustrate the effect of the raster on shearing, finite element computation based on sliced geometry is considered with a model size illustrated in Figure 11b of about 1.34 × 10^5^ elements. This method allows to obtain a complementary information of the strain localisation at the scale of the filament, which is beyond the reach of DIC approach. 

**Figure 12:**
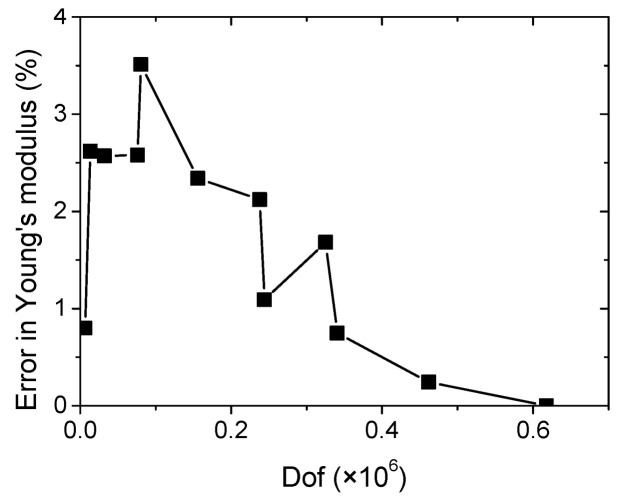
The predicted error in Young’s modulus as a function of the mesh size represented by the number of degrees of freedom (dof).

As shown in Figure 11b, the asymmetrical distribution of the generated porosity results in the inhomogeneous shear strain component ε_xy_, where X- and Y-directions are both aligned with the width and length directions of the printed specimen (Figure 11c). It is worth mentioning that the amount of shearing is limited. The most significant strain levels obviously correspond to the longitudinal strain component. The quality of this strain field is not significantly altered when the mesh size is taken above 31,995 dofs (Figure 11d). When the element size decreases, more details are added, and a better strain field description is achieved. The largest mesh size (617,703 dofs) predicts the largest contrast between the low and high strain levels. But the main characteristics of the strain field discussed earlier are preserved for meshes above 31,995 dofs. The validity of the finite element results is also checked with regard to the predicted macroscopic quantities such as Young’s modulus. This is considered by varying the number of degrees of freedom (Dof) associated to the meshed geometry. Generally speaking, the more elements are used for the meshing the higher is the accuracy of the computation and the better is the strain field description. However, fine meshes require more computation resources. So, the purpose of the mesh sensitivity considered in this study is to find the adequate balance between accuracy and robustness and to show that the predicted results are not sensitive to mesh size. In Figure 12, the plot of the error reported with respect to the largest mesh does not exceed few percent. This result shows that irregular meshing preserves the main geometrical characteristics (porosity content), which are known to influence the macroscopic quantities but the quality of the strain field proves to be more mesh dependent.

**Figure 13:**
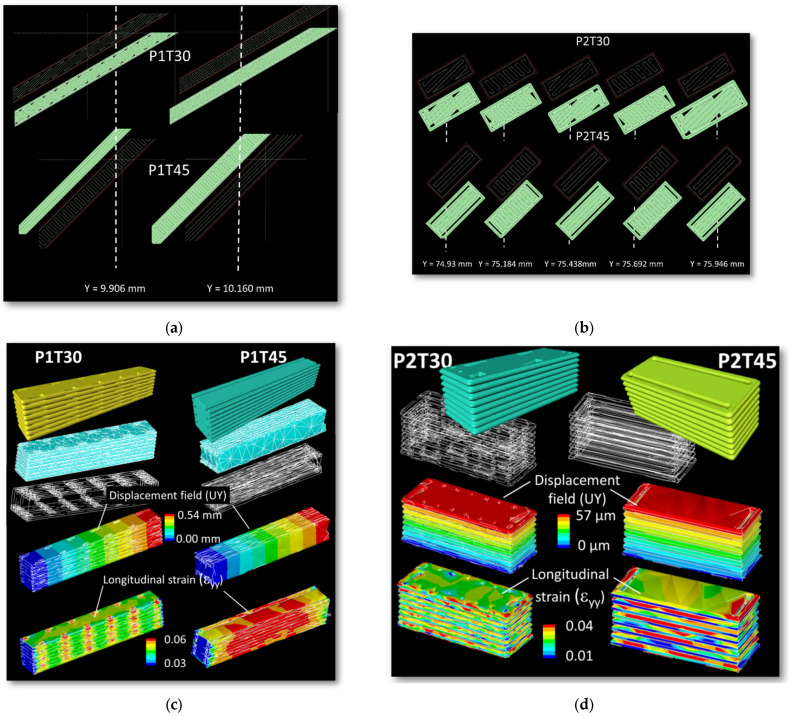
Filament trajectory along the length of the sample showing the presence of gaps between the raster and the frame as function of printing orientation: printing configurations (**a**) P1T30, P1T45, (**b**) P2T30, P2T45; (**c**,**d**) related finite element results based on the implementation of the filament trajectory for the same printing configurations showing the patterns of strain localisation.

Besides the shearing that takes place due to filling asymmetry, decohesion stands as a leading damage mechanism, which is qualitatively introduced through Figure 9b and further detailed in Figure 13. The comparison between the filament trajectories for various printing configurations P1T30 and P1T45, P2T30 and P2T45 show the presence of gaps that are generated during the filling process (Figure 13a).

In fact, these process-generated porosities create a real difficulty for FDM to match the frame generation with the filament raster. The full filling of the inner part is not achieved for all conditions, even when the raster is aligned with the main frame directions (P1T45, P2T45). The layer arrangement and part filling along the building direction (Y-direction) significantly varies depending on the printing orientation and raster angle. For the printing configuration P1 (Figure 13a), the building up is formed by a sequence of two different layers. For the printing configuration P2 (Figure 13b), the number of layers increases to four. Finite element computation shows that different patterns of strain localisation are predicted depending on the printing configuration (Figure 13c,d). This localisation triggers the unsoldering of the frame with respect to the inner part, and decohesion of the filament at the contact points is likely to occur as previously demonstrated in Figure 9b. For the printing configurations P1, the large strain levels close to the porosity percolate along the building direction. This supports the idea of preferred sites for cracking that combines with the transverse rupture of all frame filaments as illustrated in Figure 8 and Figure 10a. For the printing configurations P2, the strain localisation within the layer themselves is also influenced by the presence of the porosity near the frame (Figure 13d). Failure occurs by cracking running along the inter-layer as previously depicted in Figure 10. The starting points of cracking are formed by those locations where there is a lack of material filling as suggested by the finite element results. 

The discussion of the damage mechanisms and localisation behaviour under tension relies significantly on the understanding of the way the filaments are arranged and, on the ability, to quantify the amount and orientation of process-induced porosity. In the light of these results, the role of structural defects can be further generalised to other load configurations such as compression. Although the mechanical response is different, a recent study by the authors [42] on the compression behaviour of printed ABS shows that specific damage mechanisms are also inferred in particular arrangements of the filaments and process-induced porosity. It is easily understood that the opening of porosity and decohesion mechanisms can be mechanically captured under tension, but the most surprising result is that the same mechanisms can also prevail under compression where Poisson’s effects combined with specific arrangements of the filaments can trigger rupture under shearing mechanisms.

## 5. Conclusions

This study confirms the key role of the printing orientation compared to the raster angle in controlling the tensile performance of 3D-printed ABS. The secondary role of the raster angle is primarily due to the filament crossing sequence. The best performing configuration combines a raster angle of 0°, allowing filament crossing at −45°/+45° and a building direction normal to the loading direction. A positive effect on the elongation at break is even obtained and justified by the ability to contain damage accumulation compared to any other printing configurations. Digital image correlation demonstrates that strain localisation ranks all configurations by the extent of their strain-related localisation and bound strain levels. All component fields including longitudinal, transverse and shear strain exhibit heterogeneous fields but with spatial characteristics barely related to filament crossing logics. Finite element computation relates these observed strain patterns to the presence of stress concentrators, namely porosity close to the frame and to the asymmetrical filling within the printed structure. The simulation approach provides a complementary information about strain localisation at the scale of the filaments. SEM complemented observations show varieties of damage mechanisms encompassing inter-filament and intra-filament decohesion, frame unsoldering and layer debonding. The latter damage mechanism is the main one leading to the failure of the printed ABS polymer. The failure of worst performing configurations is linked to large, damaged areas that converge into unstable fractures.

## Figures and Tables

**Table 1:** Fixed process parameters used to manufacture FDM-based ABS specimens.

Parameter	Magnitude	Parameter	Magnitude
Material grade	P430XL ABS	Material glass transition	108 °C
Nozzle diameter	254 microns	Printing speed	0.48 cm^3^/min
Filament diameter	1.75 mm	Shell thickness	254 microns
Top/Bottom thickness	254 microns	Platform adhesion type	None
Print temperature	280 °C	Filament flow	100%
Base temperature	77 °C	Part filling	100%
Support material	Yes	Support type	Touching buildplate
Initial layer thickness	254 microns	Layer thickness	254 microns
Travel speed	150 mm/s	Cooling fan	enabled

**Table 2:** ABS sample configurations based on varied orientation (P1: parallel to sample width, P2: parallel to sample length) and raster angle θ.

Configuration	Build Orientation	Raster Angle	Filament Crossing
P1T00	P1	0°	−45°/+45°
P1T30	P1	30°	−15°/+75°
P1T45	P1	45°	0°/+90°
P2T00	P2	0°	−45°/+45°
P2T30	P2	30°	−15°/+75°
P2T45	P2	45°	0°/+90°

**Table 3:** Tensile engineering constants as a function of printing orientation and raster angle.

#	Poisson’s Coefficient (-)	Young’s Modulus (MPa)	Yield Stress (MPa)	Tensile Strength (MPa)	Ultimate Engineering Stress (MPa)	Elongation at Break (%)
P0000	0.35 ± 0.0	2310 ± 210	30 ± 0.0	34 ± 0.0	34 ± 0.0	2.93 ± 0.45
P1T00	0.37 ± 0.03	1683 ± 42	25.70 ± 0.84	27.9 ± 0.81	26.15 ± 0.17	4.0 ± 0.50
P1T30	0.29 ± 0.01	1621 ± 32	21.05 ± 0.63	23.16 ± 0.43	15.32 ± 1.7	1.9 ± 0.04
P1T45	0.37 ± 0.00	1496 ± 2	20.75 ± 0.21	23.38 ± 0.10	21.50 ± 0.5	2.0 ± 0.60
P2T00	0.37 ± 0.03	1558 ± 64	20.06 ± 1.22	21.02 ± 0.98	20.02 ± 1.48	1.5 ± 0.07
P2T30	0.37 ± 0.03	1476 ± 22	19.53 ± 0.75	19.82 ± 0.96	19.8 ± 0.97	1.5 ± 0.07
P2T45	0.28 ± 0.03	1364 ± 14	17.15 ± 0.13	17.65 ± 0.74	16.83 ± 1.83	1.5 ± 0.10

## Data Availability

Raw data are available from the authors upon request.
